# Characteristics of Selected Properties of a Medical Titanium Alloy with Nb and Zr

**DOI:** 10.3390/ma19153240

**Published:** 2026-07-31

**Authors:** Robert Dąbrowski, Krzysztof Sołek

**Affiliations:** Faculty of Metals Engineering and Industrial Computer Science, AGH University of Krakow, 30 Mickiewicza av., 30-059 Krakow, Poland; solek@agh.edu.pl

**Keywords:** medical titanium alloy, heat treatment, hardness, fracture toughness, fractographic examinations

## Abstract

**Highlights:**

- Identification of heat treatment processes of Ti13Nb13Zr alloy in order to obtain proper mechanical properties.
- Selection of optimal values of annealing and aging temperatures for improvement of fracture toughness (KIC) and hardness.
- Identifying the fracture character and its influence on the fracture toughness.
- A close relationship between the microstructure of the alloy and the obtained mechanical properties was demonstrated.

**Abstract:**

This work characterized the metallurgical properties and selected mechanical properties of the medical alloy Ti13Nb13Zr. The study included an analysis of the hardness and fracture toughness of the medical material, which was first solution heat-treated and then aged. This work significantly complements the literature on this alloy. This supplementation involved selecting previously unused heat treatment parameters: solution heat treatment from 1050 °C and subsequent aging in the range of 350 to 550 °C. After solution heat treatment from 1050 °C and quenching in water, the alloy is characterized by a microstructure composed of titanium martensite (α′) with low hardness and high fracture toughness. The applied aging process, which involves diffusion phenomena, involved the decomposition of α′ martensite and the precipitation of new α and β phases, resulting in increased hardness and decreased fracture toughness. The use of a previously unused solution heat treatment temperature of 1050 °C may additionally improve mechanical properties, including hardness and fracture toughness (K_IC_), compared to lower solution heat treatment temperatures. The results obtained, using different heat treatment conditions, were confirmed by microstructural and phase analysis. This paper presents the characteristics of the tested alloy, a description of the heat treatment, and a detailed analysis of the phase transformations occurring during aging after solution heat treatment. Considerable attention was paid to fractographic analysis of fractures in samples obtained during fracture toughness tests. The obtained results significantly expand the knowledge of the tested alloy.

## 1. Introduction

Among the many groups of metallic materials, titanium-based alloys play an important role in modern medicine. These include the so-called first-generation alloys, i.e., Commercial Pure 1, Ti6Al4V ELI, Ti6Al7Nb, and Ti5Al2Fe, and second-generation alloys: Ti12Mo6Zr2Fe, Ti35Nb7Zr5Ta, Ti29Nb13Ta4.6Zr, Ti15Mo5Zr3Al, and Ti15Mo. The latter group also includes the Ti13Nb13Zr alloy [1,2,3,4,5,6].

Compared to the iron alloys commonly used in many branches of industry, the mentioned alloys are characterized by high relative strength, high yield strength, very good fracture toughness, high fatigue strength, low Young’s modulus and good weldability [2,3,7,8,9,10]. β-rich structures, in addition to the commonly known two-phase alloys (Ti6Al4V, Ti6Al7Nb), have also found their application in the field of medicine [1,4,5,11,12]. In the mid-1990s, the chemical composition of the Ti13Nb13Zr alloy was developed. Because of the previously mentioned mechanical characteristics, excellent resistance to corrosion, and the addition of niobium (Nb) and zirconium (Zr) in its chemical composition, this material is applied in orthopedic fixation devices, including bone screws, clamps, and plates used for the stabilization of human bones [1,4,5,7,11,12,13]. Currently, attempts are being made to apply it to hip and knee joint endoprostheses, i.e., so-called long-term implants. The alloying elements (Nb and Zr) introduced into the Ti13Nb13Zr alloy (in place of aluminum and vanadium, which are added in medical, two-phase titanium alloys) are characterized by greater biocompatibility. Furthermore, their presence in the alloy’s chemical composition causes an increasing of the corrosion resistance. Passive layers containing these elements consist of Nb_2_O_5_ and ZrO_2_ oxides, which are chemically much more stable and biocompatible compared to, for example, vanadium oxide in the medical Ti6Al4V alloy. Therefore, the passive layers in the Ti13Nb13Zr alloy (unlike two-phase titanium alloys, which contain vanadium oxides in the passive layer) exhibit enough sticking to the surface and not much dissolution in the human body. The elements contained in this alloy do not cause inflammation or carcinogenic reactions [4,5,7,13]. The disadvantage of this alloy, apart from its high price, is its low tribological properties, which is why thermochemical treatment methods and coatings are commonly used [7,14,15,16,17,18,19]. Obtaining the appropriate set of features for the Ti13Nb13Zr alloy is possible by developing the right microstructure. It requires knowledge of the kinetics of phase transformations during continuous cooling and transformations during heating of the alloy [20]. In the annealed state, the alloy contains about 95% of the β phase in its microstructure with a spatially centered regular structure, which is why it has very good susceptibility to metal-forming processes. Other medical titanium alloys from the Ti-Nb-Zr system are characterized by similar good susceptibility [4,7,8,13,21,22,23,24,25]. The high addition of Nb (13%) decreases β transus temperature in relation to two-phase martensitic alloys, as well as Ms temperature and the Young’s modulus value [1,11,21,22,24]. In addition to thermo-mechanical processing, heat treatment also allows for shaping the required alloy properties [26,27,28,29,30,31]. In order to obtain the required microstructure, near β alloys are subjected to hardening and aging procedures. Heating for the hardening procedure is carried out slightly below or just above the β transus temperature, which for this alloy is 735 °C [20,32,33,34]. Cooling the alloy at a rate higher than the critical rate allows obtaining α′ or α″ martensite, similarly to two-phase titanium alloys [2,3,8,20,27,28,29,30,31,32,33,34,35,36,37,38]. In turn, slow cooling causes the diffusion transformation and separation of α and β phases from the β phase with a lamellar morphology exhibiting the features of the Widmannstätten structure or granular (globular) morphology. The aging process allows for further strength increases through martensite decomposition and the precipitation of finely dispersed α and β phases [27,28,29,30,31,32,33,34]. When the Ti13Nb13Zr alloy is slowly cooled or a high aging temperature is applied, it is also possible to obtain a hard and brittle ω phase in its microstructure. This is a phase with an orthorhombic structure, causing worst plasticity [34,39,40].

An analysis of several recent publications in the literature regarding the intended use of the Ti13Nb13Zr alloy revealed that all of them recommend its use in medicine. The publication by Hoppe et al. [12] has an experimental character. The authors investigate the properties of the Ti13Nb13Zr alloy as an implant material. The authors indicate applications including orthopedic implants, bone tissue engineering products, and dental implants. They also justify these applications with a Young’s modulus of approximately 84 GPa, high corrosion resistance in Ringer’s solution, lack of cytotoxicity towards MC3T3 and NHDF cells, and adequate mechanical properties. The authors draw these conclusions based on their own research, which supports their conclusions. The next analyzed article [41] provides a comprehensive discussion of titanium alloys used in medicine. Ti13Nb13Zr is presented as one of the most important biomedical alloys free from harmful Al and V additives. Applications include orthopedic implants, dental implants, load-bearing implants, and applications requiring good osteointegration. The authors emphasize that the alloy is valued for its lower modulus of elasticity, high biocompatibility, good corrosion resistance, and lack of toxic alloying additives. Assessing the content of this work, it should be noted that the publication does not present new applications but summarizes the state of the art. All the applications mentioned are consistent with the current literature. The next publication [42] analyzes the development of biomedical titanium alloys. Ti13Nb13Zr is presented as a classic representative of α + β alloys intended for orthopedic implants, dental implants, and components requiring a lower modulus of elasticity than Ti6Al4V. The authors also emphasize the potential of this alloy for further surface modification, 3D printing, and nanostructure development. The content of the paper reflects the current state of knowledge, but the publication is a review and does not provide new experimental evidence. Work [13] is the most detailed publication on Ti13Nb13Zr. It describes the effect of the nanostructure obtained using intense plastic deformation (SPD) methods on implant properties. The authors point to applications in orthopedic implants, bone implants, dental implants, implants requiring rapid osteointegration, and long-term implants. It is particularly emphasized that the nanostructure increases strength, improves cellular response, promotes osteogenesis, and does not compromise biocompatibility. Of the aforementioned works, this is the most comprehensive study on the practical applications of Ti13Nb13Zr. The claims are well documented in the literature. Publication [43] is not about the biology of the alloy, but rather its processing technology. Ti13Nb13Zr is listed as a biomaterial used in the production of orthopedic implants and dental implants. The authors analyze grinding difficulties, the effect of processing parameters on surface integrity, and the importance of surface quality for implant durability. This publication primarily addresses implant manufacturing and finishing technologies, not their biocompatibility or biological applications.

In the possibility of obtaining the required mechanical properties, i.e., strength, plasticity and good fracture toughness in the near β titanium alloys, the grain size of the primary β phase (D) before the hardening procedure plays an important role. Heating such alloys just above the β transus temperature causes a rapid increase in the grain size of the β phase, because there is no α phase in the microstructure which prevents growth of the β phase. In the works of [30,31] mechanical properties were investigated, including hardness and fracture toughness of the Ti13Nb13Zr alloy for annealing temperatures of 800 °C (D = 0.196 mm) and 900 °C (D = 0.289 mm) with subsequent aging at 350, 450 and 550 °C. In the alloy cooled after heating to 800 °C, 210 HV was obtained at an impact energy (KV) of 125.6 J [30]. In turn, after supersaturation at 900 °C, a higher hardness was obtained, i.e., 252 HV with lower fracture toughness (KV = 107.9 J, K_IC_ = 81.5 MPa × m^1/2^) [31]. Similarly to two-phase, martensitic titanium alloys, the application of the aging procedure at increasingly higher temperatures resulted in an increase in hardness and other strength parameters and a decrease in the fracture toughness of the Ti13Nb13Zr alloy.

The novelty of this work is an investigation of the microstructure, hardness, fracture toughness and fracture micrographs of Ti13Nb13Zr alloy samples for an annealing temperature of 1050 °C (D = 0.415 mm) with subsequent aging at 350, 450 and 550 °C. This is an extension to the research [5,23,24,25,26,27,28,29,30,31,33] and verification of the literature data on the possibility of shaping the mechanical properties of this alloy depending on the grain size of the primary β phase before the aging procedure. The application of a temperature of 1050 °C should ensure a complete α-to-β phase transformation, effective homogenization of the chemical composition, and the achievement of a metastable structure that allows for further control of properties through heat treatment, while potentially limiting the negative effects of overheating and excessive grain growth. There are few works in the available world literature in which the authors carried out heat treatment consisting of the supersaturation of titanium alloys at two different temperatures and subsequent aging, and in which the use of a higher supersaturation temperature would result in a simultaneous increase in hardness and fracture toughness. The results in this paper allow for enriched knowledge on Ti13Nb13Zr alloy characteristics, which will serve both manufacturers and users of finished elements made of this alloy.

## 2. Materials and Methods

The experimental investigations were conducted on a Ti13Nb13Zr titanium alloy classified as a near β alloy (made in China). The nominal chemical composition of the material is presented in Table 1. Quantitative analysis of alloying elements was performed by means of optical emission spectrometry.

According to the recommendations of the standard [44], the Nb content should be in the range of 13.5–14% and according to the chemical analysis it is 13.8%. The β phase is stabilized and the end temperature α + β → β change is decreased to 735 °C by this element [2,3,7,8,15,29,30,31]. In turn, the Zr content (according to the standard 13.5–14%) is 13.9%. This is a neutral element in the alloy. Its influence is to strengthen the alloy in solution and prevent precipitation in the microstructure of the brittle ω phase [2,3,7,8,36,37]. The remaining elements contained in the chemical composition are impurities. These are both α—stabilizers—i.e., N, O and C, as well as stabilizing the β phase—Fe. These elements also strengthen the analyzed alloy [2,3,5,7,8,20,36,45].

The alloy was delivered for testing in the form of a rod with a diameter of 27 mm and a length of 1000 mm in the technological state after hot deformation in the range of the existence of the β phase. Then the alloy was kept during 120 min at 700 °C before air cooling to achieve a microstructure close to β with a small amount of the α phase (approx. 5%).

In the first stage, metallographic tests of the alloy were performed. Samples with dimensions of 15 × 15 mm were cut from the longitudinal cross-section of the rod and heated at the following temperatures: 800, 850, 900, 950, 1000, 1050 and 1100 °C (in the β range) for 20 min, then cooled in water. Different supersaturating temperatures influenced the approximate size of initial β phase grains. The Jeffries procedure supported by the Saltykov correction was applied within the confines of metallographic tests. The rectangle variant was used. The stereological parameters, i.e., the average number of flat grains per surface (N_A_) and the average grain size of the primary β phase (Dav), were determined for five fields of view per one annealing temperature. For the average grain size the standard deviation was also determined.

Microstructure analysis required metal samples embedded in self-curing resin before grinding and polishing in a SiC suspension. The samples were etched in two stages, i.e., initially in a 6% HF solution and in the second stage in a solution with content similar to the Kroll reagent: 96 mL H_2_O + 2 mL HNO_3_+ 2 mL HF.

The alloy microstructure analyzed in various heat treatment states were performed using a SEM technique. A scanning electron microscope was used—the FEI Versa 3D model (FEI Company, Hillsboro, OR, USA).

In the second stage, the alloy mechanical properties were tested. Mechanical characteristics concerned the samples supersaturated from 1050 °C and then aged at 350, 450 and 550 °C during 300 min. They included hardness measurements and fracture toughness tests. The latter property was determined in static conditions (measurement of the stress intensity factor K_IC_). The hardness tests were performed using a Vickers apparatus—type HPO 250 (made in Markkleeberg, Germany, WPM Leipzig) in accordance with standards [46,47] standards. An indenter load of 10 kG (98 N) was applied. For each heat treatment variant, 8 hardness measurements were performed, from which the arithmetic mean was calculated along with the standard deviation of the mean value.

The alloy fracture toughness test was performed using the linear-elastic fracture mechanics method in a static bending test. The tests were conducted in accordance with the standard [48]. Samples with a rectangular cross-section of 20 × 10 mm and a length of 100 mm were used. A notch was mechanically cut into the samples to a depth of about 8 mm. A fatigue pre-crack with a depth of 2 mm was created at the bottom of the notches. Then, the samples were bent at three points on an INSTRON 810 computer-controlled testing machine using the “INS K_IC_” program (Instron, Norwood, MA, USA). The support span dimension during three-point bending for these specimens was 80 mm. During the tests using an extensometer, the change in the bending force was recorded as a function of the notch edge spread until the sample cracked. Based on the graphs recorded during the tests, the stress intensity factor (K_IC_) was determined using the aforementioned computer program. Three samples were tested for each variant in order to obtain sufficient precision.

Fractographic analysis of the fractures of the samples used in the K_IC_ tests were performed. They were carried out on a Nova NanoSEM 450 scanning electron microscope (SEM, FEI Company, Hillsboro, OR, USA). The observation technique used secondary electrons SE. Fractographic tests allowed for a detailed assessment of the nature of fractures after various heat treatments of the Ti13Nb13Zr alloy. They were observed at magnifications in the range of 500–5000×. In this work micrographs with 1000× magnification are presented.

## 3. Results and Discussion

### 3.1. Stereological Parameter Measurements

Table 2 presents detailed results of tests of stereological parameter of the Ti13Nb13Zr alloy microstructure (N_A_, D_av_) in the state after annealing between 800 and 1100 °C and quenching in water. Additionally, the results of the alloy hardness tests (HV 10) were also presented.

An increase in the annealing temperature of the Ti13Nb13Zr alloy from 800 to 1100 °C causes an almost 2.5-fold increase in the average grain size of the initial β phase (D_av_) from 0.186 to 0.426 mm. A similar trend is observed in single-phase β titanium alloys and in two-phase α + β alloys heated above the β transus phase transformation temperature [49,50]. Grain coarsening occurs because the β phase evolves toward minimizing grain boundary energy; this energy decreases as grains grow, since larger grains have a reduced total boundary area. In two-phase and near β alloys, below the transformation temperature, the increase in grain size (grain boundary migration) is inhibited by the α phase particles present in the microstructure. In other engineering materials, for example iron alloys, the grain size growth is inhibited by carbides not dissolved in the solution during annealing [51,52,53].

Heating the Ti13Nb13Zr alloy in the range of 800–1100 °C and subsequent cooling causes a tendency for the hardness to increase from 210 to 274 HV. This is a rather small change and the hardnesses obtained are lower than in the two-phase Ti6Al4V or Ti6Al7Nb medical alloys after cooling from the analogous temperature range [50]. Titanium martensite (α′) occurs in the microstructure of samples cooled in this way. It is a supersaturated different-node solution of elements in titanium α. The increase in the alloy hardness with decreasing of temperature is caused by the increasing supersaturation of Nb and Zr, similar to other elements (N, Fe, O, C).

### 3.2. SEM Microstructure

**Figure 1:**
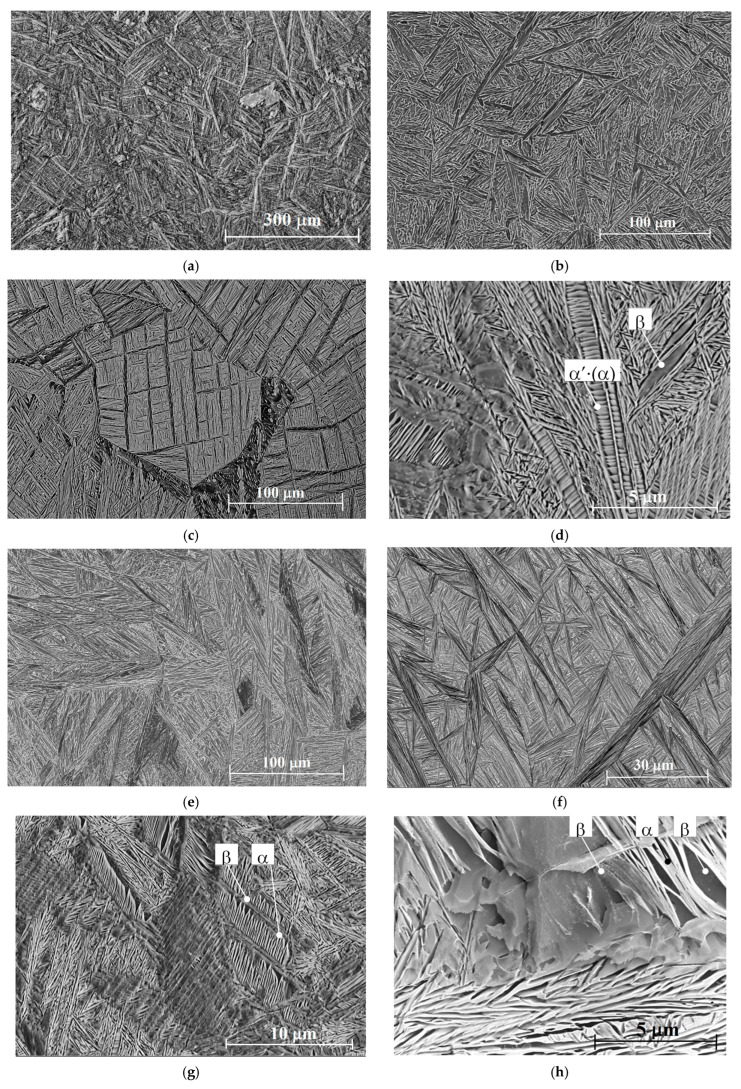
Ti13Nb13Zr microstructure developed during water quenching after soaking at 1050 °C (**a**) and subsequent aging at 350 °C (**b**), 450 °C (**c**,**d**) and 550 °C (**e**–**h**).

Figure 1 presents the Ti13Nb13Zr microstructure developed during water quenching after soaking at 1050 °C (a) and subsequent aging at 350 °C (b), 450 °C (c,d) and 550 °C (e–h).

In the case of this sample, which was only cooled, its microstructure (shown in Figure 1a) shows an acicular component, i.e., titanium martensite α′, similarly to the alloys after cooling from temperatures of 800 °C and 900 °C [30,31]. The presence in the chemical composition of β stabilizer Nb (13%) and a neutral element, Zr (13%), does not cause a decrease in temperature of the onset of the martensitic transformation Ms to below room temperature, so it is possible for a diffusionless transformation of the β phase into α′ to occur. Thus, the process of its hardening takes place, similarly to some two-phase titanium alloys, e.g., Ti6Al3Mo, Ti6Al4V, Ti6Al7Nb, Ti6Al3Mo1V, and Ti6Al2Mo2Cr [2,3,7,8,35,36,37,45,50]. The obtained component of the microstructure (martensite) is formed by plastic microdeformation of the β phase in the form of needles at an angle of 60 and 120°. In the investigated alloy cooled from 1050 °C, it is much more coarse-needled than after cooling from the temperature of 800 and 900 °C [30,31]. Its macroscopic hardness is 263 HV and is higher than after cooling from the two above-mentioned temperatures. This microstructure is also confirmed by dilatometric studies, based on the analysis of the CTPc diagram made for the Ti13Nb13Zr alloy [20]. The dilatometric curves of samples cooled at a rate of 150 °C/s (from the β range) and the corresponding differential curves show a clear positive dilatational effect (increase in the length and volume of the sample) resulting from the β → α′ martensitic transformation. It is related to the transformation of the β phase (having a smaller specific volume) into the α′ phase (having a larger specific volume). The microstructure of the alloy cooled from 1050 °C to room temperature was the starting state for further aging studies of the alloy.

Diffusion processes (350–550 °C) are based on the disintegration of the needle structure. They show some similarity to the decomposition of martensite in iron alloys, in which at low temperatures (before the precipitation of epsilon carbide or cementite), first areas of martensite enriched and depleted in carbon are formed [51,52,53]. Similarly, in titanium alloys, martensite retains high stability up to about 300–350 °C, and the material structure only contains zones with different concentrations of alloying elements—some poorer, others enriched [2,3,7,8,35,36,45]. The emergence of such regions can likely explain the minor rise in hardness observed at 350 °C (about 270 HV) compared with the quenched condition. Therefore, the application of the aging temperature of 350 °C does not lead to significant changes in the alloy microstructure (Figure 1b). Within martensitic regions that are poor in alloying constituents, the structure breaks down, leading to the development of the α phase. Alterations in internal stress levels together with a higher proportion of α′/α phase boundaries are likely responsible for the modest rise in hardness observed in the alloy. At 350 °C, the microstructure retains its acicular character. A similar situation occurs in the case of the Ti13Nb13Zr alloy aged at 350 °C after prior quenching from 900 °C [31].

Increasing the aging temperature to 450 °C causes a further increase in the hardness of the investigated alloy to 290 HV. In martensitic regions containing higher concentrations of alloying elements, a transition to the β phase takes place, as the composition of the enriched α′ phase progressively approaches thermodynamic equilibrium with rising temperature, ultimately matching that of the β phase. The alloy microstructure at 450 °C retains its acicular character (Figure 1c,d) and contains a certain volume fraction of new α and β phases. The increase in the fraction of α′/β and α/β interphase boundaries is an indirect reason for the increase in the alloy hardness. Such a microstructure also shows similarity to the microstructure obtained after soaking the Ti13Nb13Zr alloy at 900 °C and subsequent aging at 450 °C [31].

Using an aging treatment at 550 °C leads to a further rise in alloy hardness, reaching approximately 331 HV. At this temperature, an increase in the volume fraction of new precipitates from martensite of the α and β phases is observed in the microstructure. The locations of these phases in the microstructure of the investigated alloy are shown in Figure 1g,h. The higher hardness observed after aging at 550 °C can be attributed to a greater fraction of α/β phase boundaries along with rising internal stresses caused by localized lattice distortions, which stem from the coexistence of phases with different specific volumes. A similar situation occurs in the investigated alloy cooled before aging from a temperature of 900 °C [31].

### 3.3. X-Ray Diffraction

**Figure 2:**
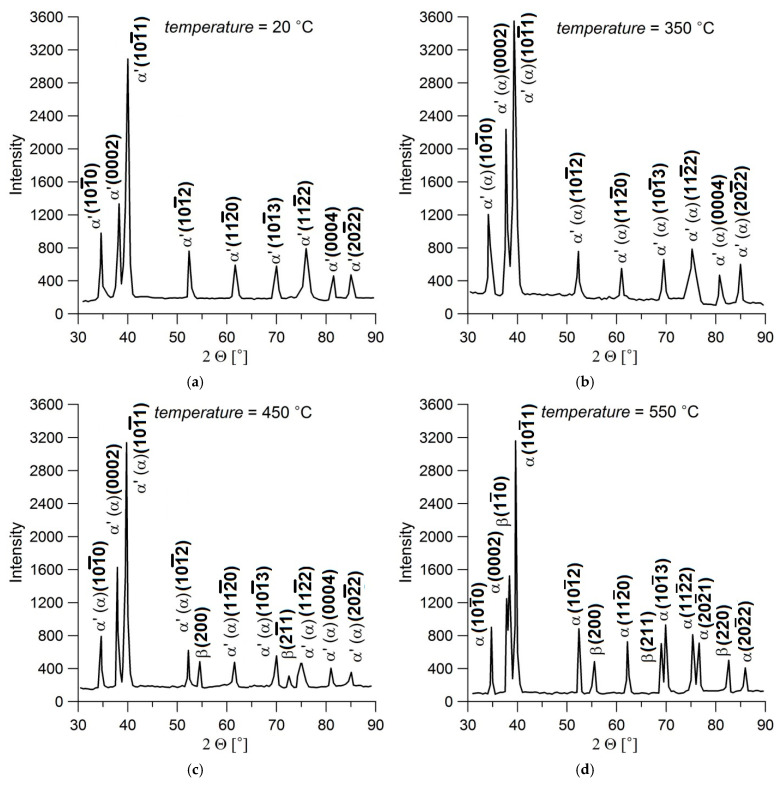
XRD plots of Ti13Nb13Zr alloy after different variants of treatment. (**a**) without aging (**b**) after aging at 350 °C (**c**) after aging at 450 °C (**d**) after aging at 550 °C.

Table 3 and Figure 2 show the influence of the aging temperature after supersaturation on the types of phases observed in the tested material (Ti13Nb13Zr). Observed phase types were determined using XRD analysis.

X-ray diffraction patterns of the Ti13Nb13Zr alloy after aging at selected temperatures and prior solution heat treatment showed the presence of reflections characteristic of the Ti-α and Ti-β phases (Figure 2). The reflections of the hexagonal Ti-α phase were assigned to the (10$\bar{1}$0), (0002), (10$\bar{1}$1), (10$\bar{1}$2), (11$\bar{2}$0), (10$\bar{1}$3), (11$\bar{2}$2), (0004), and (20$\bar{2}$2) crystallographic planes. The presence of reflections for the Ti-β phase with a space-centered cubic structure was assigned to the (110), (220), (200), and (211) planes. Changes in the reflection intensity after aging indicate the course of phase transformations associated with the decomposition of the metastable martensitic phase and the formation of α and β phase precipitates.

### 3.4. Hardness Measurements

**Figure 3:**
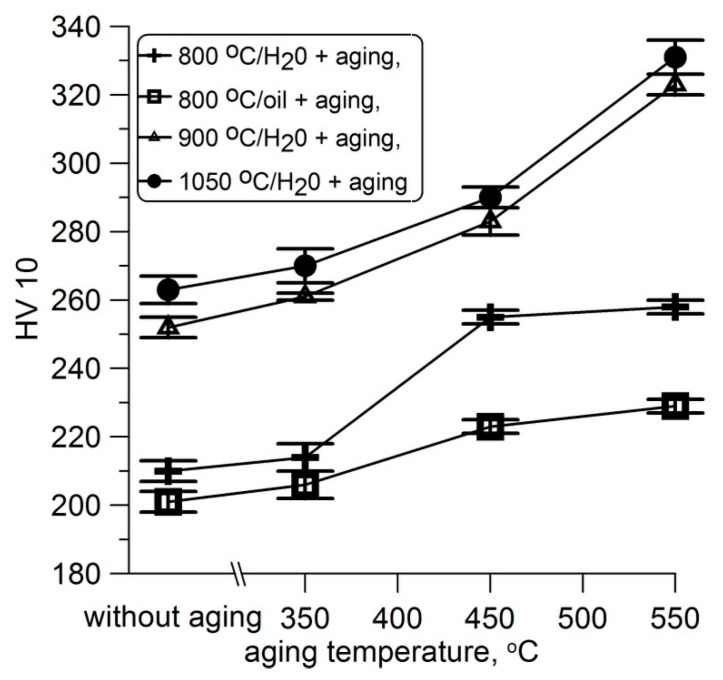
The effect of the aging temperature after solution heat treatment on the hardness of the Ti13Nb13Zr alloy. The hardness curves for 800 and 900 °C were taken from [30,31].

Figure 3 illustrates the changes in hardness of the alloy heated at 1050 °C and aged at temperatures of 350, 450 and 550 °C. Additionally, for comparative purposes, Figure 3 shows the curves of hardness changes obtained from heating at 800 °C with cooling in water and oil. In addition, an analogous curve for the heating temperature of 900 °C, taken from the work [31], is shown.

According to the literature data [2,3,45,49], the increase in hardness of the Ti13Nb13Zr alloy with increasing solution heat treatment temperature, observed in Figure 3, may be related to the formation of a more homogeneous β phase, its greater degree of supersaturation with niobium and zirconium, and increased dislocation density and internal stresses. Consequently, this leads to alloy strengthening and may increase the potential for α phase precipitation during the aging process.

The trend of increasing hardness with increasing aging temperature of the Ti13Nb13Zr alloy, observed in Figure 3, and the mechanisms responsible for this phenomenon, are discussed in detail in Section 3.2. Generally, the increase in hardness is associated with the gradual decomposition of titanium martensite, formed during cooling after solution heat treatment, and the precipitation of fine particles of the α and β phases.

These observations are also confirmed in [28,54] concerning the effect of the aging process on the microstructure and mechanical properties of the Ti13Nb13Zr alloy. In [54], a significant effect of aging time on the mechanical properties of the tested alloy was demonstrated. Increasing the aging time from 10 min to 6 h resulted in increased hardness, mechanical strength, and Young’s modulus. Simultaneously, increasing hardness and strength led to a gradual decrease in the ductility of the Ti13Nb13Zr alloy. A similar pattern of hardness changes during aging is also observed in other biomedical β-type titanium alloys [55,56]. In [55], it was demonstrated that during aging at temperatures between 400 and 600 °C, following solution annealing of the metastable β phase, the α phase precipitates, resulting in increased hardness and strength of the Ti34Nb2Ta0.5O alloy. The authors of [56], while studying the microstructure and properties of the Ti28Nb13Zr0.5Fe alloy, found that during aging at 350 °C, the β phase is first precipitated into the metastable ω phase, and then, in the temperature range of 450–550 °C, the α phase is precipitated. The presence of these precipitates in the microstructure results in a significant increase in the hardness of the tested alloy.

### 3.5. Fracture Toughness Measurements

Table 4 presents the detailed results of the alloy hardness measurements for each heat treatment variant. This table also contains a detailed summary of parameters obtained in the static three-point bending test of the Ti13Nb13Zr alloy samples. These parameters were as follows: average value of the maximum force acted on the sample (*F_max_*), total average notch length (*a_average_*), and quotient of average notch length to sample height (*a_average_*/*h*). The above parameters were used to determine the stress intensity factor K_IC_, i.e., the basic measure of the resistance of the tested alloy to crack propagation. It should be emphasized that the K_IC_ parameter measurements, in accordance with the [48] standard, were conducted under plane strain conditions. In this condition, the internal stresses causing crack propagation in the tested alloy were the lowest.

Analysis of the hardness test results for the Ti13Nb13Zr alloy indicates an increase in this parameter from 263 HV (in the hardened state from a temperature of 1050 °C) to 331 HV (in the state after aging at 550 °C). This is therefore an increase in hardness by approx. 20%. The standard deviation of the average hardness value does not exceed 5 Vickers units. It should be noted that analogous changes are observed after its hardening from 800 and 900 °C [30,31]. Titanium martensite, i.e., the metastable and transitional phase, is increasingly supersaturated with alloying elements with increasing of the hardening temperature. A similar trend of changes was shown in β titanium alloys (supersaturated from an increasingly higher annealing temperature) [3,7,21,22,38,52,57]. In addition, two-phase martensitic alloys and near β alloys, when water-quenched from temperatures below and above the phase transformation (β transus), are also characterized by an increase in hardness after following aging process [3,8,35,36,37,45,50,58].

Aging of titanium martensite in the Ti13Nb13Zr alloy causes a decrease in its fracture toughness (K_IC_). The application of increasingly higher aging temperature gives a similar effect as in the Ti13Nb13Zr alloy cooled from 900 °C [31]. The stress intensity factor K_IC_ decreases from 78.6 MPa × m^1/2^ (in the only cooled state) to 61.9 MPa × m^1/2^ (in the state after aging at 550 °C). Thus, this is a decrease in the fracture toughness of the alloy by about 17%. The value of the maximum force loading the sample (*F_max_*) during the K_IC_ test ranges from 7.05 to 10.80 kN. The minimum value is achieved for material treated at 550 °C. In turn, the results of total, average notch length range from 9.82 to 10.98 mm (*a_av_*/*h* = 0.49–0.55).

For the Ti13Nb13Zr alloy under study, the observed variations in fracture toughness under both static and dynamic testing result from the progressive decomposition of the martensitic microstructure and the formation of new α and β phases as the aging temperature rises.

**Figure 4:**
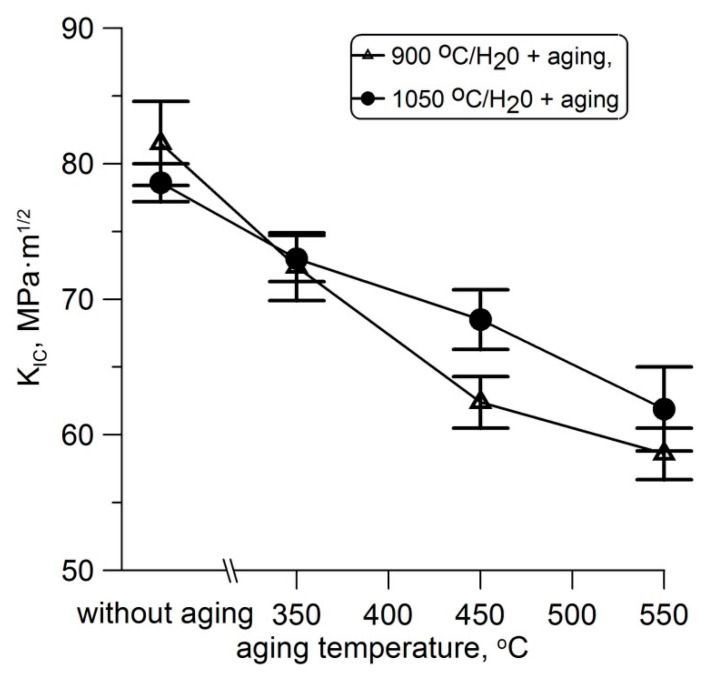
The relationship between the KIC parameter and the aging temperature in the Ti13Nb13Zr alloy samples after supersaturated from 900 °C and 1050 °C. The stress intensity factor values for 900 °C were taken from [31].

Figure 4 graphically presents the dependence of the stress intensity factor K_IC_ of the Ti13Nb13Zr alloy on the annealing temperature applied before aging (900 °C and 1050 °C). The results for the 900 °C supersaturating temperature were taken from [31].

Toughness of fracture for two-phase martensitic systems, previously quenched from the α + β → β transformation region, also diminishes as the aging temperature rises [45]. For example, the Ti6Al3Mo1V alloy in a state only cooled from the transformation range is characterized by a K_IC_ parameter of 56.3 MPa × m^1/2^. The application of the aging procedure (550 °C) at an increasingly higher temperature causes a gradual decrease in the K_IC_ parameter to 34.2 MPa × m^1/2^ [45]. Conversely, β-type titanium exhibits a comparable reduction in factor of stress intensity as the aging temperature rises following supersaturation treatment. It concerns, for example, the medical alloy Ti24Nb4Zr8Sn, for which the decrease in the impact energy of aged samples is caused by the small precipitates of α phase with compact hexagonal structure from supersaturated β phase [59].

The Ti13Nb13Zr alloy solution annealed from 1050 °C and aged in the temperature range of 350 to 550 °C is characterized by higher K_IC_ values compared to the alloy solution annealed from a lower temperature of 900 °C (Figure 4). This may be due to several factors. Ti13Nb13Zr is a β-type titanium alloy, sometimes referred to in the literature as a near β alloy. The β transus temperature for this alloy is approximately 735 °C. Both 900 °C and 1050 °C are above this temperature, but provide different initial conditions. Annealing at the higher temperature of 1050 °C produces a larger volume of homogeneous β phase before quenching, which allows for more complete dissolution of the alloying elements (Nb, Zr). This more homogeneous microstructure often promotes better fracture toughness. Furthermore, if the material was hardened from 1050 °C, the α-phase precipitates during aging may be more uniformly distributed, finer, and less susceptible to crack propagation. As a result, higher fracture toughness can be achieved despite the higher hardness of this alloy. There are publications on biomedical titanium alloys that show that fracture toughness after aging can be higher when the alloy was previously solution heat-treated from a higher temperature, but this depends on whether the higher temperature leads to a favorable morphology of the α-phase precipitates or causes excessive grain growth of the β phase [54,60,61,62]. Therefore, in biomedical β-titanium alloys, the simple relationship of decreasing fracture toughness with increasing solution heat treatment temperature does not hold. For example, in [60] on the fracture toughness of the biomedical Ti29Nb13Ta4.6Zr alloy, it was shown that fine, homogeneous α-phase precipitates increase fracture toughness. Its appropriate morphology can effectively inhibit crack propagation. The paper does not compare several solution heat treatment temperatures, but it demonstrates a mechanism by which a higher solution heat treatment temperature can lead to greater fracture toughness if fine α precipitates form during aging. However, in [61], the authors observed that for the same alloy, at low aging temperatures, a brittle ω phase forms, while at higher aging temperatures, a fine α phase dominates. Mechanical and fatigue properties are more favorable for the microstructure with the fine α phase than with the ω phase. Available studies of biomedical β-Ti alloys (especially Ti29Nb13Ta4.6Zr) have shown that fracture toughness is strongly dependent on the morphology of the α phase precipitates formed during aging. A higher solution temperature may promote a more homogeneous β solution and finer α precipitates after aging, which creates conditions for improved fracture toughness. However, direct comparisons of different solution temperatures with respect to the K_IC_ factor in biomedical titanium alloys are currently rare. Therefore, this paper adds to the existing knowledge on the effect of different solution temperatures on fracture toughness after aging, which will be a significant novelty in the literature.

**Figure 5:**
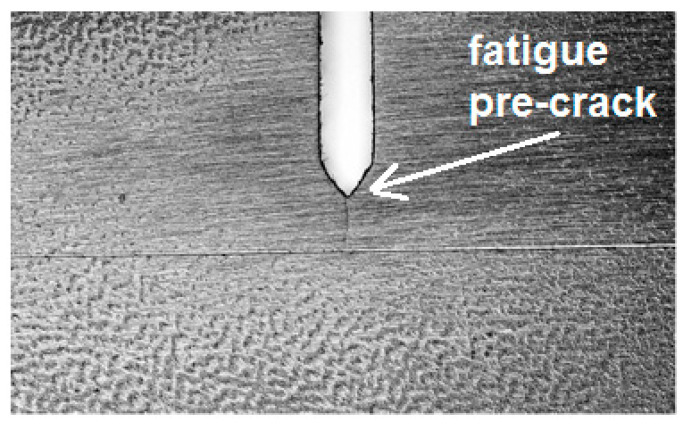
A view of the central part of the sample used for K_IC_ tests with the previously generated fatigue crack.

**Figure 6:**
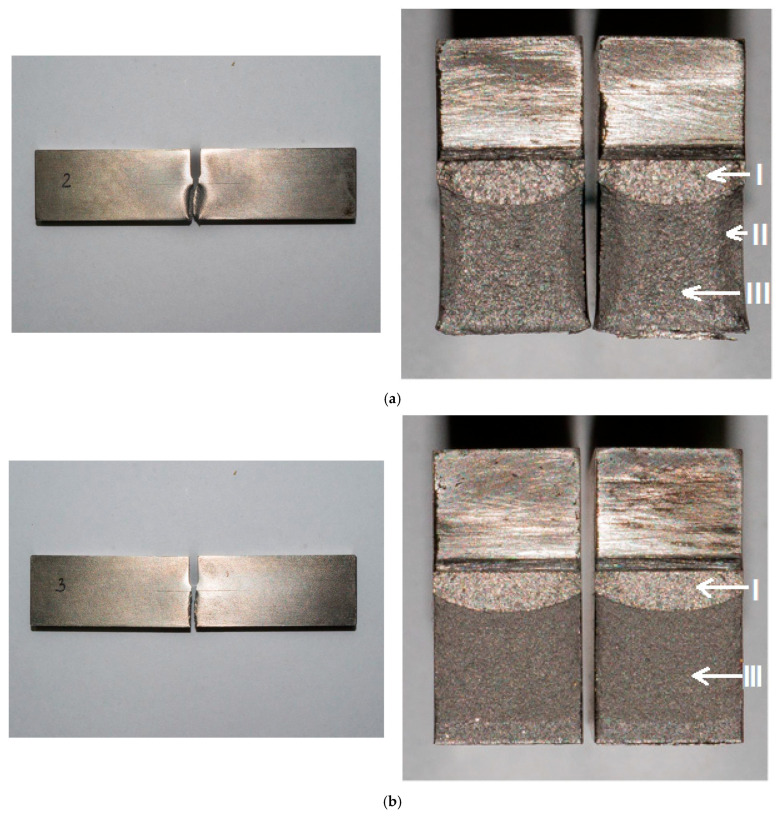
Photographs of an example sample of the Ti13Nb13Zr alloy; (**a**) aged at 350 °C and (**b**) aged at 450 °C, after the K_IC_ test (**left column**) and a macrograph of its fracture (**right column**).

Figure 5 shows a macrograph of an example sample used for testing of the stress intensity factor (K_IC_), in which a characteristic fatigue pre-crack was formed at the notch base. Figure 6 show macrographs of example samples after performing the static test of bending based on three points (K_IC_).

Figure 6a shows a macrograph of a fracture of an example sample aged at 350 °C. Based on the analysis of macrophotographs (Figure 6a), in heat-treated samples subjected to the K_IC_ test, three areas should be distinguished. Area I—this is a bright area with a clear boundary of the end of the fatigue pre-crack generation. Area II—occurring on the sides of the sample (matte color) characteristic of places of plastic deformation, where the edge parts of the sample were broken at an angle of approx. 45°. Area III—central, more brittle, and shiny, in relation to the edges of the sample in area II. In area III, individual grains are clearly visible.

It should be noted that a similar macrofracture pattern was observed in samples of the Ti13Nb13Zr alloy only cooled from 1050 °C. Clear traces of plastic deformation were observed at the edges of these samples.

A different macrofracture pattern is visible in the sample aged at 450 °C after cooling from 1050 °C (Figure 6b). A characteristic area with a fatigue pre-crack is visible. The middle region shows a marked reduction in grain size relative to the specimen aged at 350 °C (see Figure 6a). Plastic deformation was not observed at the edges of the specimens after aging at 450 °C. It should be noted that a similar macrofracture pattern appeared in specimens previously aged at 550 °C. It appears that a lack of plastic strain along the specimens’ edges aged at 450 and 550 °C played a role in lowering their K_IC_ measurements (refer to Table 4).

### 3.6. SEM Fractography

**Figure 7:**
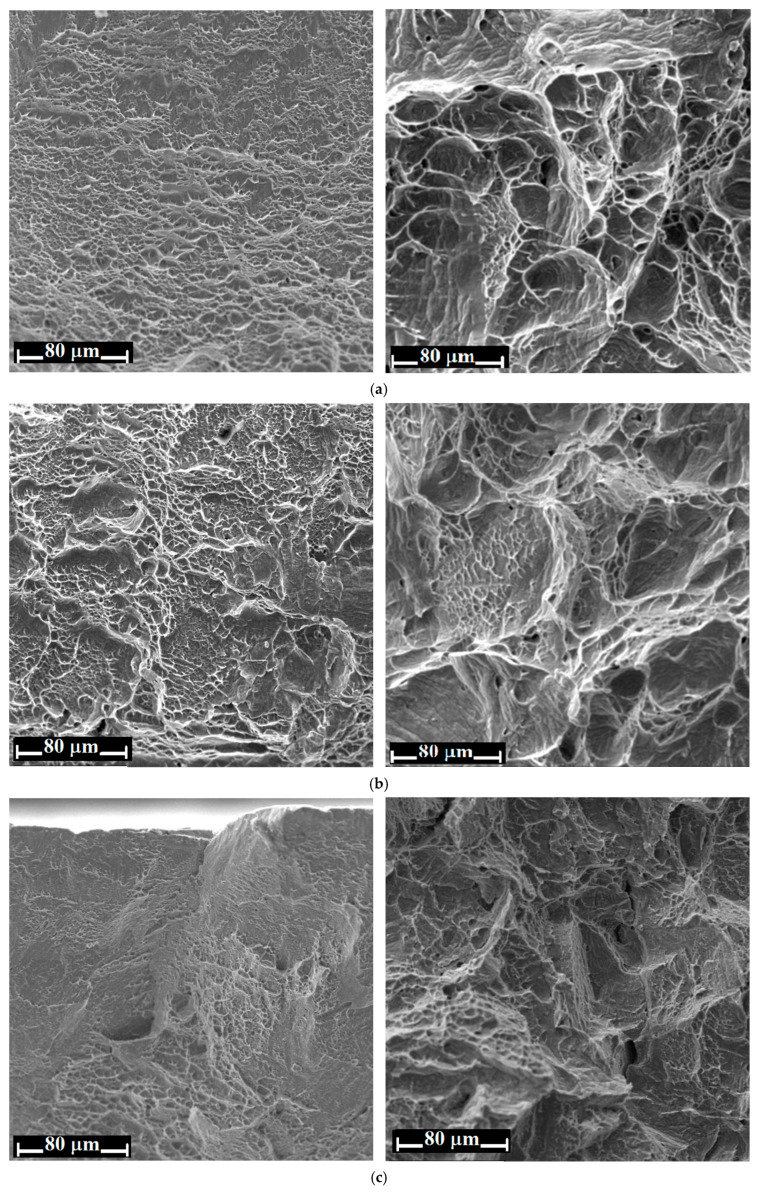

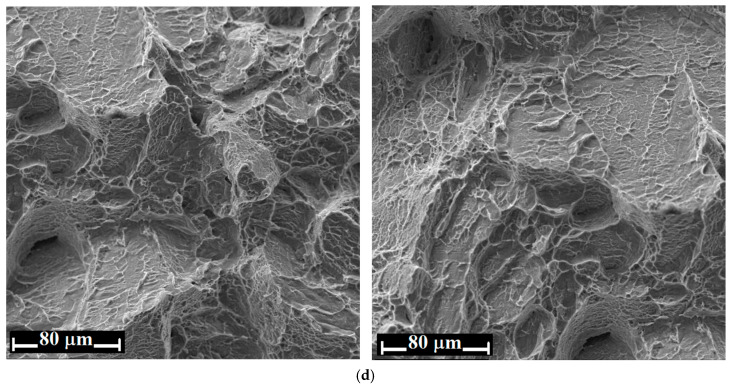
SEM observations of the Ti13Nb13Zr specimens subjected to K_IC_ parameter estimation after heat treatment: (**a**) after supersaturating at 1050 °C, (**b**) after aging at 350 °C, (**c**) after aging at 450 °C, (**d**) after aging at 550 °C—following an earlier cooling stage from 1050 °C (**left column**—fracture from the edge part of the sample; **right column**—fracture from the central part).

The Ti13Nb13Zr fracture toughness data (see Table 4) are supported by the observed alterations in the fracture character of the specimens tested for K_IC_. These fractures, observed with use of a scanning electron microscope (SEM), are shown in Figure 7. Figure 7 (left column) presents fractures obtained in the edge part of the samples, while Figure 7 (right column) presents those obtained in their central part.

The fractures of Ti13Nb13Zr alloy samples (used for testing the K_IC_ parameter) presented in Figure 7a (left column) show a different character depending on the heat treatment method. They are typical for near β alloys and β alloys subjected to saturation heat treatment with subsequent aging [3,5,9,22,38,63].

In the case of the alloy in the hardened condition (Figure 7a (left column)) or hardened and low-aged condition (Figure 7b (left column)), a ductile dimple fracture is visible in the edge parts of the specimens. In the case of the specimen aged at 350 °C, a certain share of brittle fracture is observed, which is practically not visible in the sample only cooled from 1050 °C. In the central part of the aforementioned samples (Figure 7b,c—right column), a mixed fracture is also visible, with an increase in the share of brittle fracture compared to the edge parts of the specimens. For the specimen aged at the lower temperature of 350 °C, hardness rises while fracture toughness shows a modest reduction (refer to Table 4). This could be due to the transformation of the material from the α′ structure into the α form. The properties change occurred as a result of thermal effects associated with aging at a temperature of 350 °C. Probably, the gradual disintegration of martensite results in a decrease in the degree of supersaturation with alloying elements, but at the same time it results in an increase in the share of α′/α interphase boundaries. A decrease in the degree of supersaturation would result in a decrease in hardness, but an increase in the share of interphase boundaries may cause the opposite effect. The reduction in fracture toughness of the Ti13Nb13Zr alloy at 350 °C (Table 4) might be related to a lower share (and lower development) of the plastic fracture surface, especially near the specimen’s edge after aging at 350 °C (Figure 7b—left column).

The specimen fractures after aging in 450 °C and 550 °C, shown in Figure 7c,d, show a different character. These are mixed fractures. They show the features of a brittle fracture with a small share of dimpled areas. This phenomenon could suggest that precipitate formation promotes transgranular cracking in the investigated alloy. Analysis of phase transformations between 450 and 550 °C, along with the disintegration of martensite, suggests that this is associated with the early formation of finely dispersed β phase precipitates. The stress intensity factor (after aging at 450 °C,) was recorded at the level of 68.5 MPa × m^1/2^. This factor is lower than for the specimen after aging at 350 °C (see Table 4). Further raising the temperature of the aging process (up to 550 °C) results in a rise in the alloy hardness to 331 HV and a slight decrease in fracture toughness (see Table 4). It results in a change in the nature of fractures, which are largely brittle and contain a much smaller proportion of ductile areas compared to the sample aged at 450 °C (see Figure 7c,d).

The micrographs of sample fractures presented in Figure 7 only qualitatively illustrate the changes in their nature, depending on the heat treatment parameters used for the Ti13Nb13Zr alloy. The observed images are qualitatively consistent with the obtained fracture toughness values (K_IC_ parameter) resulting from the applied heat treatment (see Table 4).

**Figure 8:**
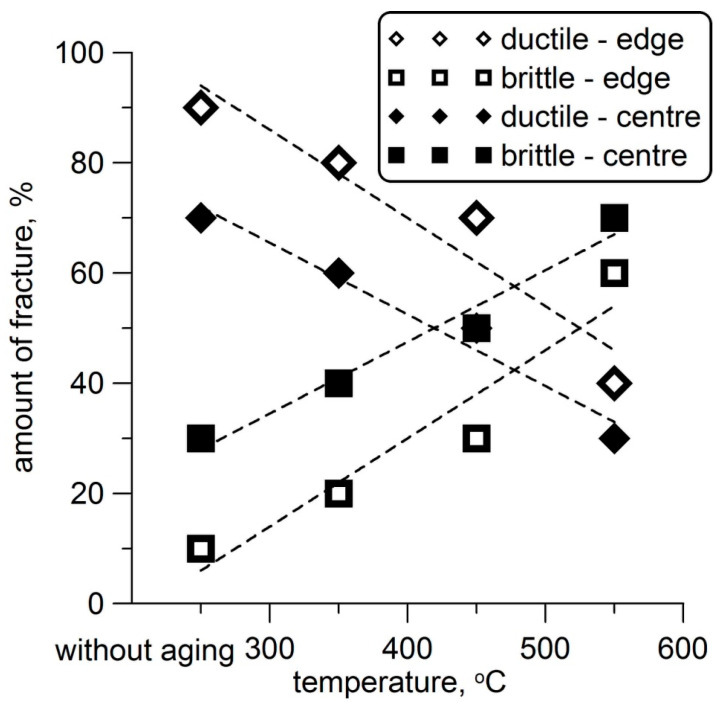
Changes in the proportion of ductile and brittle fracture in the edge and central part of heat-treated Ti13Nb13Zr alloy samples.

Table 5 and Figure 8 present the test results quantitatively illustrating the changes recorded in microfractures of the heat-treated samples used for stress intensity factor (K_IC_) testing. Based on the analysis of microfractures from the samples’ edge and central areas, the proportion of ductile and brittle fractures was determined. This analysis showed that with increasing aging temperature after solution heat treatment, the volume fraction of brittle fractures increases and the proportion of ductile regions decreases. This trend is maintained in both microfractures from the samples’ edge and central areas (Table 5 and Figure 8). The nature of the changes is also consistent with the qualitative analysis.

Table 6 summarizes the obtained test results for the Ti13Nb13Zr alloy after the heat treatment proposed by the authors, consisting in solution annealing from a temperature of 1050 °C and aging in the range of 350–550 °C.

## 4. Summary and Conclusions

This article characterizes selected mechanical properties of the medical titanium alloy Ti13Nb13Zr after the proposed heat treatment, which included solution heat treatment from 1050 °C and aging in the temperature range of 350–550 °C. A detailed analysis of the changes occurring in the alloy’s microstructure and their impact on hardness and fracture toughness is presented. The conducted research led to the following summary and conclusions:

1. After solution heat treatment from 1050 °C and quenching in water, the tested alloy is characterized by a microstructure composed of titanium martensite (α′). In this state, it exhibits a relatively low hardness of 263 HV, while the fracture toughness (K_IC_) reaches 78.6 MPa × m^1/2^.
2. Aging in the temperature range of 350–550 °C, during which diffusion processes occur, causes an increase in hardness to 331 HV and a simultaneous decrease in fracture toughness to 61.9 MPa × m^1/2^ after aging at 550 °C. These changes are associated with the gradual decomposition of martensite and the precipitation of new α and β phases, the presence of which was confirmed by X-ray diffraction (XRD).
3. With increasing aging temperature, a systematic decrease in the stress intensity factor K_IC_ is observed. This phenomenon was confirmed both by observations of macrocracks in the samples used for fracture toughness testing and by detailed analysis of the fractures performed using scanning electron microscopy (SEM).
4. Samples cooled in water from 1050 °C and aged at 350 °C exhibited a ductile fracture with a small proportion of brittle regions. Macrocrack observations also revealed the presence of pronounced plastic deformation at the edges of these samples. However, samples aged at 450 and 550 °C exhibited a mixed fracture, with a dominant brittle fracture and a small number of ductile regions. No clear traces of plastic deformation were observed at the edges of these samples, which may be the reason for the lower K_IC_ parameter.
5. Data obtained in this work could suggest that use of a higher solution heat treatment temperature (1050 °C) followed by aging of the Ti13Nb13Zr alloy could lead to greater hardness and fracture toughness compared to solution heat treatment from 900 °C. A higher solution heat treatment temperature can promote the complete dissolution of the α phase and a more uniform distribution of stabilizing elements, i.e., niobium and zirconium, in the titanium matrix. However, upon cooling from 900 °C, closer to the β transus temperature (735 °C), locally undissolved α phase may remain in the microstructure and lead to heterogeneity in the chemical composition. The uniform microstructure obtained after solution heat treatment from 1050 °C may favor the precipitation of fine and uniformly distributed α phase particles during aging, which can more effectively inhibit crack propagation, contributing to increased fracture toughness.
6. A qualitative and quantitative relationship between the solution heat treatment temperature and the grain size of the original β phase was established. The obtained results indicate that the grain size of this phase after solution heat treatment at 1050 °C (D = 0.415 mm) probably cannot adversely affect the alloy’s fracture toughness after final aging. This could be confirmed by the higher K_IC_ values and increased hardness compared to that samples that were solution heat-treated from 900 °C.
7. The heat treatment parameters used in this work for the Ti13Nb13Zr alloy, including supersaturation from 1050 °C and aging in the range of 350–550 °C, have not been widely reported in the literature. The obtained results can indicate that the proposed heat treatment method effectively shapes the mechanical properties of this alloy, leading to a favorable combination of high hardness and good fracture toughness. The presented qualitative and quantitative results regarding the mechanical parameters have significant scientific and cognitive value and also indicate the application potential of the developed solution.

## Figures and Tables

**Table 1:** The chemical composition (wt. %) of the Ti13Nb13Zr alloy.

Element	Ti	Nb	Zr	Fe	O	N	C
ASTM F1713	bal.	13.5–14.0	13.5–14.0	max 0.05	max 0.04	max 0.02	max 0.10
Analysis	bal.	13.8	13.9	0.040	0.040	0.010	0.06

**Table 2:** The impact of heat treatment temperature during annealing on both the characteristic grain size of the initial β phase (N_A_, Dav) in the Ti13Nb13Zr alloy and the resulting hardness measured after the cooling stage.

Annealing Temperature	Annealing Time	Cooling Conditions	N_A_	D_av_/σ	HV10
[°C]	[min]	-	mm^−2^	[mm]	-
800	20	H_2_O	20.7	0.186/0.016	210
850	20	H_2_O	15.8	0.237/0.019	233
900	20	H_2_O	11.5	0.289/0.035	252
950	20	H_2_O	8.0	0.338/0.031	255
1000	20	H_2_O	6.0	0.373/0.033	257
1050	20	H_2_O	4.4	0.415/0.042	263
1100	20	H_2_O	2.6	0.429/0.044	274

**Table 3:** The effect of the aging temperature after supersaturation on the types of phases observed in the tested material (Ti13Nb13Zr).

Variants	Parameters of Aging		Supersaturating 1050 °C/1 h/H_2_O
	Temp.	Time	
	°C	h	Observed Phases
I	-	-	α′
II	350	5	α′ (α)
III	450	5	α′ (α) + β
IV	550	5	α + β

**Table 4:** A comprehensive overview is provided of hardness and fracture toughness measurements for the Ti13Nb13Zr alloy following heat treatment at 1050 °C for 60 minutes, water quenching, and subsequent aging at 350, 450, and 550 °C for 300 minutes.

Aging Parameters		Solution Treatment Conditions—1050 °C/1 h/				
Temp.	Time	F_max_	a_av_	a_av_/h	K_IC_	HV10
°C	h	kN	mm	-	MPa × m^1/2^	kG/mm^2^
-	-	10.80	9.82	0.49	78.6	263
350	5	9.13	10.35	0.52	73.0	270
450	5	8.84	10.19	0.51	68.5	290
550	5	7.05	10.98	0.55	61.9	331

**Table 5:** A summary of the quantitative parameters describing microfractures from the edge part and central part of heat-treated Ti13Nb13Zr alloy samples.

Parameter	Unit	Solution Treatment Condition—1050 °C/1 h/H_2_O			
		Aging Parameters			
		Without	350 °C/5 h/Air	450 °C 5 h/Air	550 °C 5 h/Air
Edge part of sample					
Ductile fracture	%	85–95	75–85	65–75	35–45
Brittle fracture	%	5–15	15–25	25–35	55–65
Central part of sample					
Ductile fracture	%	65–75	55–65	45–55	25–35
Brittle fracture	%	25–35	35–45	45–55	65–75

**Table 6:** Summary of investigation results obtained for heat treatment proposed by authors.

Parameters of Aging		Supersaturating 1050 °C/1 h/H_2_O				
Temp.	Time	Observed Phases	HV10	K_IC_	Ductile Fracture	Brittle Fracture
					Central Part of Sample	
°C	h		kG/mm^2^	MPa × m^−1/2^	%	%
-	-	α′	263	78.6	65–75	25–35
350	5	α′ (α)	270	73.0	55–65	35–45
450	5	α′ (α) + β	290	68.5	45–55	45–55
550	5	α + β	331	61.9	25–35	65–75

## Data Availability

The original contributions presented in this study are included in the article. Further inquiries can be directed to the corresponding author.
